# Serotonin 5-HT_2C_ receptor knockout in mice attenuates fear responses in contextual or cued but not compound context-cue fear conditioning

**DOI:** 10.1038/s41398-022-01815-2

**Published:** 2022-02-11

**Authors:** Youcef Bouchekioua, Mao Nebuka, Hitomi Sasamori, Naoya Nishitani, Chiaki Sugiura, Masaaki Sato, Mitsuhiro Yoshioka, Yu Ohmura

**Affiliations:** 1 Department of Neuropharmacology, Faculty of Medicine and Graduate School of Medicine, Hokkaido University, Sapporo, Japan; 2 Laboratory of Molecular Pharmacology, Institute of Medical, Pharmaceutical and Health Sciences, Kanazawa University, Kanazawa, Japan

**Keywords:** Learning and memory, Psychiatric disorders

## Abstract

Previous findings have proposed that drugs targeting 5-HT_2C_ receptors could be promising candidates in the treatment of trauma- and stress-related disorders. However, the reduction of conditioned freezing observed in 5-HT_2C_ receptor knock-out (KO) mice in previous studies could alternatively be accounted for by increased locomotor activity. To neutralize the confound of individual differences in locomotor activity, we measured a ratio of fear responses during versus before the presentation of a conditioned stimulus previously paired with a footshock (as a fear measure) by utilizing a conditioned licking suppression paradigm. We first confirmed that 5-HT_2C_ receptor gene KO attenuated fear responses to distinct types of single conditioned stimuli (context or tone) independently of locomotor activity. We then assessed the effects of 5-HT_2C_ receptor gene KO on compound fear responses by examining mice that were jointly conditioned to a context and a tone and later re-exposed separately to each. We found that separate re-exposure to individual components of a complex fear memory (i.e., context and tone) failed to elicit contextual fear extinction in both 5-HT_2C_ receptor gene KO and wild-type mice, and also abolished differences between genotypes in tone-cued fear extinction. This study delineates a previously overlooked role of 5-HT_2C_ receptors in conditioned fear responses, and invites caution in the future assessment of molecular targets and candidate therapies for the treatment of PTSD.

## Introduction

Posttraumatic stress disorder (PTSD), a trauma- and stress-related disorder, develops after experiencing a traumatic event. With a worldwide lifetime prevalence of 3.9% [1], PTSD has become a global health issue and is characterized by excessive physiological arousal and dysregulated fear responses to contexts and cues previously associated with the traumatic event. Selective serotonin reuptake inhibitors (SSRIs) alleviate these symptoms partially, although approximately 40% of SSRI-treated patients are non-responders. Serotonin 5-HT_2C_ receptor gene KO in mice or 5-HT_2C_ receptor antagonist administration have been found to reduce anxiety-related behaviors [2–6], suggesting that serotonin 5-HT_2C_ receptors could be a promising target in the treatment of PTSD.

Pavlovian fear conditioning has been extensively used as a relevant model of PTSD [7]. Intraperitoneal (i.p.) injection of the 5-HT_2C_ receptor antagonist SB242085 (0.3 mg/kg) in rats reduced freezing behavior in contextual fear conditioning [6]. Consistent with Ohyama et al. (2016) [6], we recently assessed the freezing behavior of 5-HT_2C_ receptor KO mice in contextual fear conditioning and found that they exhibited a rapid within-session extinction, although there was no difference in retrieval and between-session extinction of fear memory compared to WT control mice [4]. However, freezing behavior, which is defined as the absence of movement except for respiratory-related movements [7], can be affected by altered levels of locomotor activity. We have recently shown that 5-HT_2C_ receptor KO mice display an increased locomotor activity in both zero-maze and open-field tasks [4]. The reduction of freezing in 5-HT_2C_ receptor KO mice or mice treated with 5-HT_2C_ receptor antagonists could thus alternatively be explained by their higher locomotor activity trait.

Moreover, according to associative learning theories, the intensity of fear responses to stimuli can be affected when multiple conditioned stimuli (CSs) previously paired with the same aversive event interact with each other [8–11]. Indeed, it has been shown that a reduction in fear responses to a cue is associated with sustained contextual fear responses when cue-specific and contextual information are combined during conditioning but separately presented during testing [12–14]. Underestimating cue-competition effects may thus lead to an incomplete evaluation of potential PTSD therapeutics, such as those targeting 5-HT_2C_ receptors.

The aim of the current set of experiments was twofold. We first attempted to neutralize the confounding factor of locomotor activity while assessing the effect of 5-HT_2C_ receptor gene KO in fear responses. Second, we aimed to assess the role of 5-HT_2C_ receptors in fear responses to a single CS (i.e., the context in Experiment 1; a tone in Experiment 2) paired with a foot shock as an aversive unconditioned stimulus (US), or to two CSs (context + tone in Experiment 3) paired together with the US but extinguished in separate sessions. In Experiment 1 and 2, we used a conditioned lick suppression procedure where water-deprived mice are presented with a single CS (the context or a tone) previously paired with a US, while they consumed water. Retrieval of the CS-US fear memory is expected to suppress water consumption, thereby reducing the number of licks during the CS presentation. We calculated a ratio of the number of licks before versus during the CS presentation, thereby neutralizing the potential effect of locomotor activity in fear responses. In Experiment 3, cue-specific (i.e., tone) and contextual information were embedded during conditioning but extinguished separately to test whether this procedure affects fear responses. In other words, we tested whether 5-HT_2C_ receptor gene KO is still effective in the compound situation.

## Methods and materials

### Animals

We used 5-HT_2C_R KO male mice (RRID:IMSR_JAX:015821) [15, 16], C57BL/6 N WT male littermates, and C57BL/6 N WT male mice supplied from Nippon SLC Co. Ltd (Hamamatsu, Japan). 5-HT_2C_ receptor KO mice and their littermates were backcrossed to the C57BL/6 N strain for more than ten generations. All mice were aged >56 days prior to the start of the experiment. Animals were kept in rooms under an alternating light-dark cycle (light from 7 p.m. to 7 a.m.) at 25 ± 2 °C and a relative humidity of 40–60%. All experimental sessions were performed during the dark period. The treatment of animals complied with the Guidelines for the Care and Use of Laboratory Animals of the Animal Research Committee of Hokkaido University, and all procedures were approved by the Animal Research Committee of Hokkaido University (approval no. 18-0070).

### Behavioral procedures

#### Apparatus

**Fig. 1:**
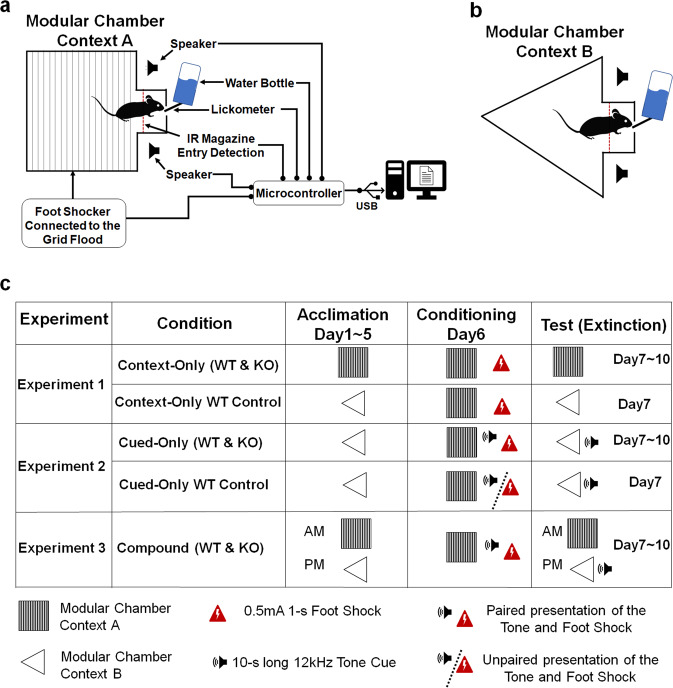
Schematic representation of the experimental setup and procedures. **a** Schematic drawing of a mouse drinking water inside the magazine of a modular chamber (Context B). The foot shock output was connected to the grid floor. The foot shock input, two speakers, the infrared (IR) magazine entry sensor, and the lickometer were connected to a microcontroller. The microcontroller was connected to a microcomputer via USB cable. **b** Schematic representation of the modular chamber arranged in Context A, with a white plastic foam sheet covering the grid floor and a triangular-shaped transparent acrylic ceiling. **c** Experimental flowchart for each condition (in Rows). Columns represents the three different phases of the procedure (Acclimation, Conditioning, and Test).

All experiments were conducted in two identical modular chambers (lab-hacks.com, France, 18 × 28 × 31 cm^3^) containing a 3 cm-wide magazine illuminated by a warm white LED at 350 mcd of typical luminous intensity (Adafruit LED Sequins). Chambers were composed of a grid floor made from stainless steel rods separated by a 0.6 cm gap. The floor and ceiling features could be manipulated to create a second distinct context (Context B). In Context B, the roof was of triangular shape and the grid floor was fully covered by a white plastic panel with a rough surface (Fig. 1). In Context A, the triangular roof and white plastic floor were removed. Foot shocks could be delivered to the grid floor by a precision animal shocker (H13-15, Coulbourne Instruments, Harvard Apparatus). The foot shocks, two speakers, and the LED were controlled by a microcontroller (Arduino Mega2560, Arduino, Italy). A 12 kHz pure tone could be delivered via the two speakers at 80 dB (C-scale). Magazine entries were detected by an infrared break beam sensor (Adafruit, IR Break Beam Sensor – 5 mm LEDs) placed at the entrance of the magazine around the water port level height and were connected to the microcontroller. The number of licks were detected during the acclimation and contextual fear extinction phases by the microcontroller using a sensor (MPR121-12, Adafruit, US, NY) connected to the spout of the water bottle via a processed wire. All data were sent from the microcontroller to a microcomputer (Raspberry Pi, UK) through a USB cable. A custom-written python script was used to collect and save data in a comma-separated values (CSV) file.

#### Experiment 1: Contextual conditioned lick suppression

After mice were handled for 5 min for at least 3 consecutive days, water bottles were removed from their home cage 24 h prior to the beginning of the experiment. The next day, mice in the Context-Only condition received 5 days (Day 1-5) of acclimation to the conditioning chambers (in Context A for WT and KO mice; in Context B for WT Control mice) (Fig. 1). Five-day acclimation was used to ensure that mice reached a steady rate of licking performance (Supplementary Fig. 1). During this phase, mice had free access to the water-filled lick tubes. Mice were given free access to water for 30 min in their home cage after each session throughout the whole experiment. We excluded any mouse that failed to drink from the water-filled lick tubes on Days 1 and 2. On Day 6, all mice in the Context-Only condition were presented six times with a 1-s, 0.5-mA foot shock in Context A. Magazine entries and access to the water-filled lick tubes were prevented by an opaque black plastic sheet placed in front of the magazine. The onsets of the shock occurred at 5.15, 9.15, 13.15, 17.15, 21.15, and 25.15 min into the session. On Days 7 to 10, WT and KO mice in the Context-Only condition were tested for contextual conditioned lick suppression in Context A, while WT Control mice in the same condition were tested only on Day 7 in Context B. To validate our procedure, WT Control mice were acclimated and tested in Context B, while they received contextual fear conditioning in Context A on Day 6. We expected that fear responses of WT mice would be higher than that of WT Control mice, which would suggest that fear responses of WT mice are a consequence of the retrieval of the Context A-foot shock memory. The absence (or at least low level) of fear responses in WT Control mice would also ensure that WT mice performance is not due to contextual fear generalization (i.e., WT mice fear responses are Context A-specific). A contextual conditioned lick suppression test session lasted 30 min and was similar in every aspect to an acclimation session.

#### Experiment 2: Cued conditioned lick suppression

The acclimation phase for mice in the Cued-Only condition was similar in every aspect to that of WT Control mice in the Context-Only condition. On Day 6, WT and KO mice in the Cue-Only condition were presented six times with a 10-s long 12 kHz pure tone cue co-terminated with a 1-s 0.5-mA foot shock in Context A. For WT Control mice in the same condition, the six presentations of the tone cue and the foot shock were unpaired. Magazine entries and access to the water-filled lick tubes were prevented by an opaque black plastic sheet placed in front of the magazine. The onsets of the tone occurred at 5, 9, 13, 17, 21, and 25 min into the session for WT and KO mice. For WT Control mice, the onsets of the foot shock occurred at 8.5, 11.7, 15.9, 18.3, 22.25, and 23.8 min into the session. On Days 7 to 10, WT and KO mice in the Cue-Only condition were tested for cued conditioned lick suppression, while WT Control mice in the same condition were tested only on Day 7. To validate our procedure, WT Control mice received unpaired presentations of the tone and the foot shock during the cued fear conditioning phase on Day 6, while the number of presentations of the tone and foot shock was the same as for WT mice. We expected that fear responses of WT mice would be higher than that of WT Control mice, which would suggest that fear responses of WT mice are a consequence of the retrieval of the tone-foot shock memory. The absence (or at least low level) of fear responses in WT Control mice would also ensure that WT mice learned the predictive value of the tone, due to the temporal contiguity of the tone and the foot shock during the conditioning phase. For all mice in the Cued-Only condition, a cued conditioned lick suppression test consisted of a 30 min session in Context B, during which five cumulative seconds inside the magazine triggered the 10-s tone cue alone. After the offset of the latter, five cumulative seconds inside the magazine triggered the tone cue again, up to 5 times. The test session ended if 5 tone cues were triggered, or after 30 min elapsed.

#### Experiment 3: Compound context-cue conditioned lick suppression

To examine the effects of cue competition in our paradigm, we used a compound of two CSs (context and tone) paired together with a foot shock, but extinguished in separate sessions. All mice in the Compound condition received two sessions of acclimation per day. In the morning, the acclimation session was the same as that of WT and KO mice in the Context-Only condition. In the afternoon, the second acclimation session was the same as that of WT Control mice in the Context-Only condition. Day 6 for WT and KO mice in the Compound condition was similar in every aspect to that of WT and KO mice in the Cued-Only condition. On Days 7 to 10, all mice in the Compound condition were tested for contextual conditioned lick suppression in the morning, and cued conditioned lick suppression in the afternoon (6 h later). The contextual conditioned lick suppression test consisted of the same test procedure as that of WT and KO mice in the Context-Only condition, and the cued conditioned lick suppression test was similar in every aspect to the test procedure used in WT and KO mice in the Cued-Only condition.

### Data analysis

To neutralize individual differences in locomotor activity and water consumption, we calculated a conditioned lick suppression ratio [17] as a measure of fear response calculated as follows:

$${{{\mathrm{Conditioned}}}}\;{{{\mathrm{Lick}}}}\;{{{\mathrm{Suppression}}}}\;{{{\mathrm{Ratio}}}} = \frac{{{{{\mathrm{Baseline}}}} - {{{\mathrm{Performance}}}}}}{{{{{\mathrm{Baseline}}}} + {{{\mathrm{Performance}}}}}}$$

For the contextual conditioned lick suppression ratio, the Baseline score refers to averaged rates of licks measured during the 30-min sessions of the two last days of the acclimation phase (i.e., Days 4 and 5), and the Performance score refers to the rates of licks measured during the 30-min sessions of the test phase (i.e., Days 7 to 10).

For the cued conditioned lick suppression ratio, the Baseline score refers to rates of licks during the 5-s period that precedes the onset of the tone cue, and the Performance score refers to rates of licks during the first 5 s of the tone cue. A low or negative score indicates a low or an absence of conditioned lick suppression (i.e., conditioned fear response), respectively, while a high score indicates a strong conditioned lick suppression.

Data were analyzed using SPSS 23.0 (IBM, NY, USA) and plotted with Prism (GraphPad, San Diego, USA). The sample size (N) was decided based on previous reports using the same type of experiments [18, 19]. No randomization was used to determine how animals were allocated to experimental groups. No blinding was done because all the data were collected automatically. An unpaired Student’s t-test for independent samples was used for two-group comparisons, with Bonferroni correction when required. A two-way analysis of variance (ANOVA) for repeated measures followed by the Bonferroni post-hoc test was used for multiple comparisons. If Mauchly’s sphericity test was significant, a Greenhouse-Geisser correction was used. A log-rang (Mantel-Cox) test was used when comparing the survival rate performance between groups in the cued conditioned lick suppression task. Data are expressed as means ± SEM, except for the survival rate where probabilities of survival are presented.

## Results

### Experiment 1: 5-HT_2C_ receptor KO mice displayed reduced contextual conditioned fear responses compared to WT mice

**Fig. 2:**
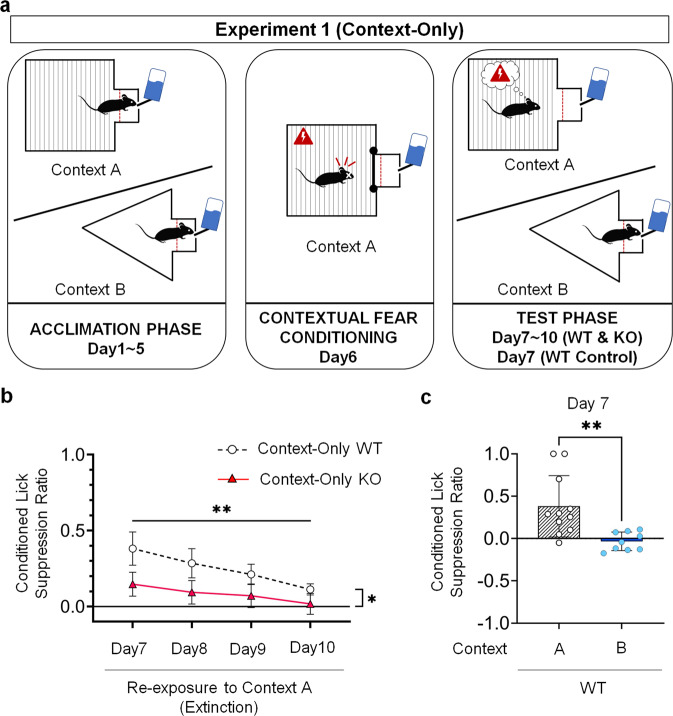
Contextual conditioned fear responses. **a** Schematic drawing of each of the three phases of the contextual condition lick suppression task. **b** Contextual conditioned lick suppression performance in WT mice (Context-Only WT Group; *N* = 11) compared to KO mice (Context-Only KO Group; *N* = 11) tested from Day 7 to Day 10 in the same context (Context A) where they received the fear conditioning session. Two-way analysis of variance (ANOVA) for repeated measures, followed by Bonferroni post-hoc test. Data are expressed as means and error bars represent ±SEM. Asterisks indicate *P*-values (*P < 0.05, **P < 0.01). **c** Conditioned lick suppression performance in WT mice tested at Day 7 in the same context as the conditioning context (Context-Only WT Group; *N* = 11) compared to WT mice tested at Day 7 in a different context (Context B) than the conditioning context (Context-Only Control WT Group; *N* = 9). Two-tailed t-test for independent samples with a Bonferroni correction. Data are expressed as means and error bars represent ±SEM. Asterisks indicate *P*-values (**P < 0.01).

When tested in the same context where contextual fear conditioning was conducted (Context B), KO mice (Context-Only KO Group, *N* = 11) showed a significantly lower conditioned lick suppression ratio when compared to WT mice (Context-Only WT Group, *N* = 11) (Fig. 2a, b) as revealed by a two-way ANOVA for repeated measures (*F*(1, 10) = 7.369, *p* = 0.022). Mice of both genotypes extinguished contextual fear responses across sessions of the test phase, as suggested by a statistically significant decrease in conditioned lick suppression ratio across extinction sessions (*F*(1.539, 15.390) = 5.219, *p* = 0.005, with a Greenhouse-Geisser correction). No statistically significant interaction between groups and extinction days was found (*F*(1.603, 16.030) = 0.720, *p* = 0.473, with a Greenhouse-Geisser correction). These data indicate that KO mice manifested reduced contextual fear responses compared to WT mice, but extinguished their fear responses at the same rate.

As a validation of the contextual conditioned lick suppression task used here, we ran an additional group of WT mice (Context-Only Control WT, *N* = 9) acclimated and tested in a context (Context A) that differs from that in which they received contextual fear conditioning (Context B). Their performance on the first day of extinction (Day 7) was compared with that of mice from the Context-Only WT Group on the same day. The higher ratio observed in the Context-Only WT Group (Mean = 0.3810, SD = 0.3621) compared to the Context-Only Control WT Group (Mean = −0.0334, SD = 0.1093) was statistically significant as determined by a two-tailed t-test for independent samples with a Bonferroni correction (*t*(12.162) = 3.601, *p* = 0.004) (Fig. 2c). These results confirmed that the conditioned fear responses observed in the Context-Only WT Group were a consequence of the Context B→Foot shock memory formed during the contextual fear conditioning phase and retrieved during the test phase. These data validate the use of contextual conditioned lick suppression as a measure of fear responses to a context previously paired with an aversive event.

### Experiment 2: 5-HT_2C_ receptor KO mice display reduced cued conditioned fear responses compared to WT mice

**Fig. 3:**
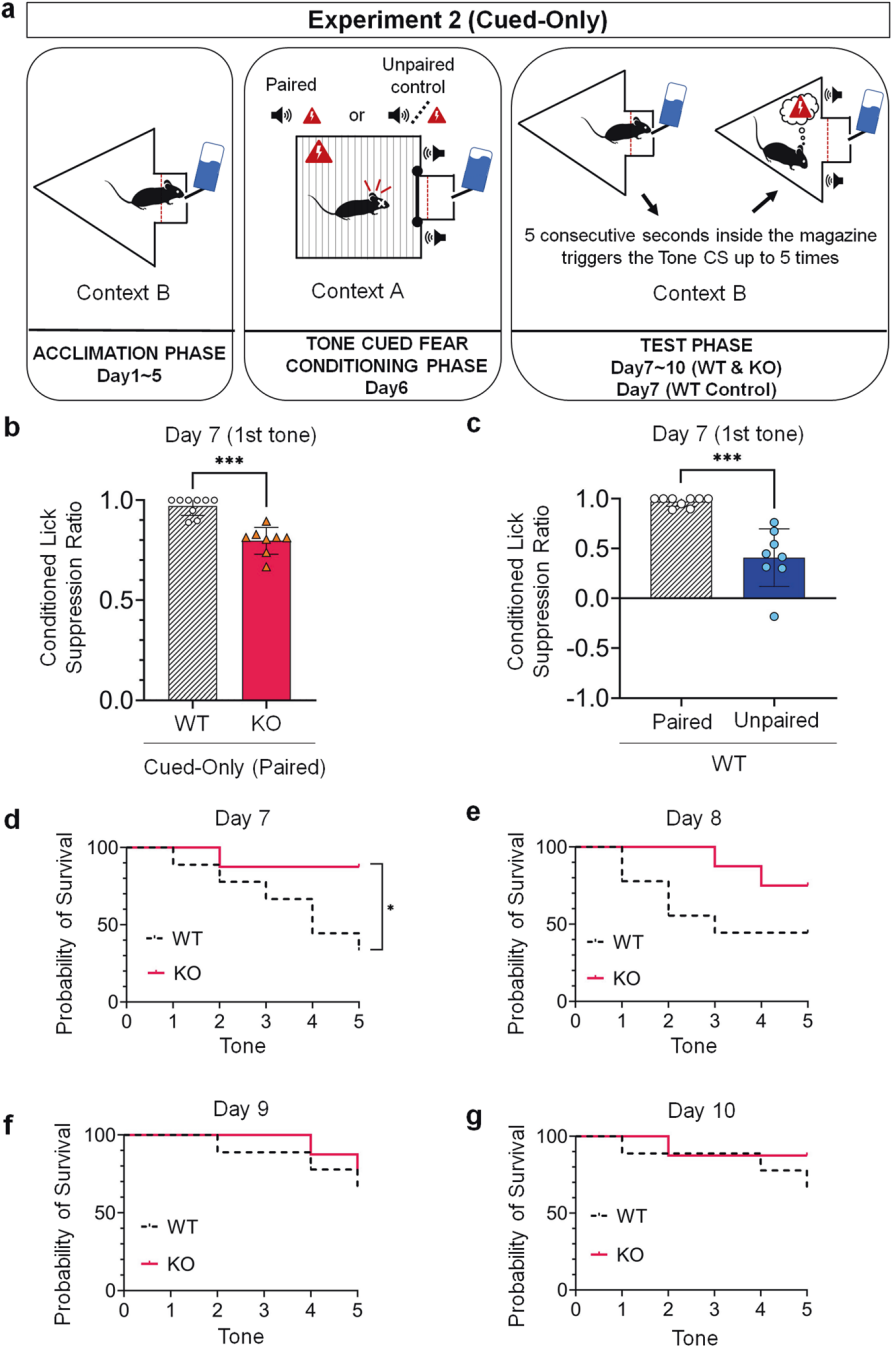
Cued conditioned lick fear responses. **a** Schematic drawing of each of the three phases of the cued condition lick suppression task. **b** Cued conditioned lick suppression performance in WT mice (Cued-Only WT Group; *N* = 9) compared to KO mice (Cued-Only KO Group; *N* = 8) during the first tone delivery at Day 7, in a different context (Context B) from that of the conditioning phase (Context A). **c** Cued conditioned lick suppression performance during the first tone delivery at Day 7 in WT mice that received paired presentations of the tone and footshock during the conditioning phase (Cued-Only WT Group; *N* = 9) compared to WT mice that received unpaired presentation of the tone and footshock during the conditioning phase (Cued-Only WT Control Group; *N* = 8). Both groups where conditioned in Context A and tested in Context B. Two-tailed t-test for independent samples with a Bonferroni correction. Data are represented as means and error bars represent ±SEM. Asterisks indicate *P*-values (***P < 0.001). **d–g** Percentage probability of survival rate between WT (Cued-Only WT Group; *N* = 9) and KO mice (Cued-Only KO Group; *N* = 8) tested from Day 7 to Day 10. Log-rank (Mantel-Cox) tests. Asterisks indicate *P*-values (*P < 0.05).

We first compared the cued conditioned lick suppression ratio of mice during the first tone delivery at Day 7 (i.e., Extinction Day 1) and found a lower suppression ratio in the Cue-Only KO Group (*N* = 8, Mean = 0.7968, SD = 0.0674) compared to the Cue-Only WT group (*N* = 9, Mean = 0.9706, SD = 0.0470), an effect that was statistically significant as determined by a two-tailed *t-*test for independent samples with a Bonferroni correction (*t*(15) = 6.228, *p* < 0.001) (Fig. 3b). These data indicate that KO mice manifest reduced cued fear responses compared to WT mice during the first tone presentation.

As a validation of the contextual cued lick suppression task used here, we ran an additional group of WT mice (Cue-Only Control WT, *N* = 8) similar in every aspect to mice from the Cue-Only WT Group, except for the conditioning phase where mice from the Cue-Only Control WT Group received unpaired presentations of the tone and foot shock. We found a significantly lower cue-conditioned suppression lick ratio in mice from the Cue-Only Control WT mice (Mean = 0.4087, SD = 0.2887) compared to mice from the Cue-Only WT Group (Mean = 0.9706, SD = 0.0470) as determined by a two-tailed *t*-test for independent samples with a Bonferroni correction (*t*(7.330) = 5.440, *p* = 0.001) (Fig. 3c). This result validates the use of cued lick suppression as a measure of fear responses to a tone cue previously paired with an aversive event. It is worth noting that all mice from the Cue-Only Control WT Group triggered the 5 tones during the test session (i.e., 100% of probability of survival; data not shown).

To further analyze cued fear responses within each extinction session, we compared the percentage probability of survival rate between genotypes and found a significantly lower score in mice from the Cue-Only WT Group compared to mice from Cue-Only KO Group only on the first day of extinction (Day 7: *χ*^2^ = 4.36, *p* = 0.037; Day 8: *χ*^2^ = 2.21, *p* = 0.014; Day 9: *χ*^2^ = 0.19, *p* = 0.66; Day 10: *χ*^2^ = 0.88, *p* = 0.35) (Fig. 3d–g). These results further strengthen the conclusion that KO mice manifest reduced fear responses to the tone cue, as they were less impacted by it compared to WT mice.

During cued conditioned lick suppression tests, five consecutive seconds spent inside the magazine triggered the delivery of the tone cue. The latter could be triggered up to five times each session. Thus, mice were naïve to these contingencies only for the first tone delivery at Day 7 (i.e., Extinction Day 1), which consists of a Pavlovian conditioning test. However, mice might have learned along with subsequent trials that their behavior (i.e., entering the magazine for five cumulative seconds) triggers the delivery of a tone previously paired with a shock (Fig. 3a). As a consequence, we can expect that mice learn to avoid entering the magazine at the early stages of the test phase and start entering the magazine again as the extinction procedure progresses, consistent with the percentage probability survival rate (Fig. 3d–g). In addition to the survival rate, we examined cued fear extinction for each day by averaging the conditioned lick suppression ratios measured for all tones triggered. As mice did not always trigger the five tones, we used a mixed-effects analysis of variance (ANOVA) and found that mice of both genotypes extinguished cued fear responses across sessions of the test phase, as suggested by a statistically significant decrease in conditioned lick suppression ratio across extinction sessions (*F*(3, 24) = 7.22, *p* = 0.001) (Supplementary Fig. 2). No statistically significant difference between genotype or no statistically significant interaction between groups and extinction days was found.

### Experiment 3a: separate re-exposure to cue- and context-conditioned stimuli prevents contextual fear extinction in 5-HT_2C_ receptor KO and WT mice and abolishes genotype differences in contextual fear responses

**Fig. 4:**
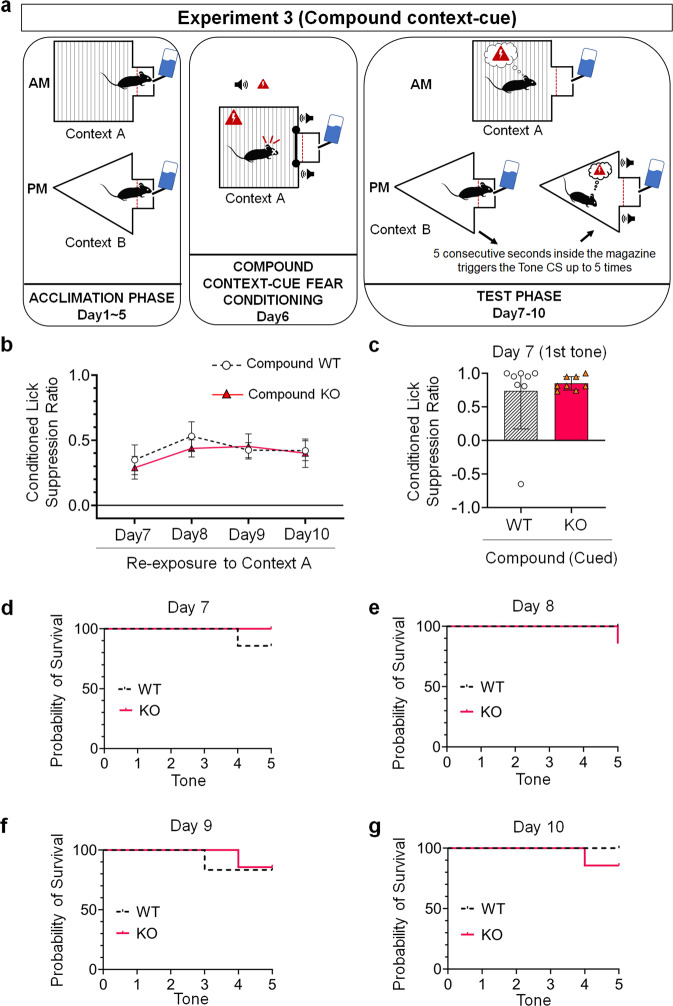
Compound context-cue fear responses. **a** Schematic drawing of each of the three phases of the compound context-cue condition lick suppression task. **b** Contextual conditioned lick suppression performance in WT mice (Compound WT Group; *N* = 7) compared to KO mice (Compound KO Group; *N* = 7) tested from Day 7 to Day 10 in the same context (Context A) where they received the fear conditioning session. Two-way analysis of variance (ANOVA) for repeated measures. Data are represented as means and error bars represent ±SEM. **c** Cued conditioned lick suppression performance in WT mice (Compound WT Group; *N* = 7) compared to KO mice (Compound KO Group; *N* = 7) during the first tone delivery at Day 7 in a different context (Context B) from that of the conditioning phase (Context A). Two-tailed t-test for independent samples. Black full lines and dashed black lines of violin plots represent medians and quartiles (first and third) respectively. **d–g** Percentage probability of survival rate between WT (Compound WT Group; *N* = 7) and KO mice (Compound KO Group; *N* = 7) tested from Day 7 to Day 10. Log-rank (Mantel-Cox) tests.

We further examined whether 5-HT_2C_ receptor gene KO exerts the same effects on fear responses even when re-exposing cued and contextual information separately (Fig. 4a). The analysis of re-exposure of contextual information using a two-way ANOVA for repeated measures revealed no statistically significant difference between WT (Compound-Context WT Group, *N* = 7) and KO mice (Compound-Context KO Group, *N* = 7) in the Compound condition (*F*(1.000, 6.000) = 0.288, *p* = 0.611), and that the conditioned suppression ratio did not differ significantly between extinction days (*F*(2.014, 12.083) = 1.508, *p* = 0.260) (Fig. 4b). There was no statistically significant genotype*extinction interaction (Greenhouse-Geisser correction) (*F*(3, 18) = 0. 492, *p* = 0.692). These data indicate that both KO and WT manifested a similar level of contextual conditioned lick suppression and highlight a deficit of contextual fear extinction in the Compound condition.

### Experiment 3b: compound context-cue fear conditioning abolishes differences in cued fear responses in 5-HT_2C_ receptor KO and WT mice

Re-exposing mice to cued information also resulted in a different consequence. We first compared the cued conditioned lick suppression ratio of mice during the first tone delivery at Day 7 (i.e., Extinction Day 1) and found that the conditioned lick suppression ratio in the Compound-Cue KO Group (*N* = 7, Mean = 0.8376, SD = 0.1003) compared to that of the Compound-Cue WT group (*N* = 7, Mean = 0.7240, SD = 0.6098) was not different as determined by a two-tailed *t-*test for independent samples (*t*(12) = −0.486, *p* = 0.636) (Fig. 4c). These data indicate that both genotypes manifested similar cued fear responses during the first tone presentation in the Compound condition.

To further analyze cued fear responses within each extinction session, we compared the percentage probability of survival rate between genotypes and did not find any differences between the Compound-Cue WT and Compound-Cue KO Groups for each day of the test phase (Day 7: *χ*^2^ = 1.00, *p* = 0.32; Day 8: *χ*^2^ = 2.17, *p* = 0.14; Day 9: *χ*^2^ = 0.003, *p* = 0.96; Day 10: *χ*^2^ = 1.00, *p* = 0.32) (Fig. 4d–g). These results further strengthen the conclusion that both genotypes manifested similar fear responses to the tone cue.

In addition to the survival rate, we examined cued fear extinction for each day by averaging the conditioned lick suppression ratios measured for all tones triggered. As mice did not always trigger the five tones, we used a mixed-effects analysis of variance (ANOVA) and found that mice of both genotypes extinguished cued fear responses across sessions of the test phase, as suggested by a statistically significant decrease in conditioned lick suppression ratio across extinction sessions (*F*(3, 35) = 4. 71, *p* = 0.007) (Supplementary Fig. 2). No statistically significant difference between genotypes and no statistically significant interaction between groups and extinction days were found.

## Discussion

Prior reports showed a reduction of freezing responses in 5-HT_2C_ receptor gene KO mice [20] or after 5-HT_2C_ receptor antagonist administration [6], but these findings could alternatively be explained by increased locomotor activity in these animals. To resolve this problem, we used a conditioned licking suppression paradigm and clearly demonstrated that genetic deletion of 5-HT_2C_ receptors in mice attenuates fear responses in contextual or cued conditioning independent of changes in locomotor activity. (Figs. 2 and 3). To our knowledge, the present study is the first to control for the effects of locomotor activity while assessing the role of 5-HT_2C_ receptors in contextual fear responses. The procedures developed here should prove useful in exploring potential therapeutics for fear-related symptoms, especially when using animal models with different baselines of locomotor activity compared to WT animals.

Previous studies have demonstrated that the amygdala is necessary for cued fear while the hippocampus is necessary for contextual fear [21] and that 5-HT_2C_ receptors are expressed in both regions [22, 23]. A previous study showed that stress increased the expression of 5-HT_2C_ receptors in the amygdala, and the pharmacological blockade of 5-HT_2C_ receptors in the amygdala attenuated fear responses to a tone cue previously paired with a footshock [24]. Thus, it is likely that the reduction of cued fear response in 5-HT_2C_ receptor KO mice (Fig. 3) is due to the lack of 5-HT_2C_ receptors in the amygdala. In accordance with this hypothesis, our previous study showed that the pharmacological blockade of 5-HT_2C_ receptors in the ventral hippocampus did not reduce contextual fear response in rats [25]. It should be noted that we injected the antagonist immediately before the re-exposure to examine the role of 5-HT_2C_ receptors in the retrieval of contextual fear memory. Moreover, we previously found that 5-HT_2C_ receptor KO mice displayed a slower acquisition of fear conditioning [4]. Thus, it is possible that the reduction of contextual fear response in 5-HT_2C_ receptor KO mice (Fig. 2) is due to a deficit in the acquisition of contextual fear conditioning rather than the retrieval of contextual fear memory. Alternatively, 5-HT_2C_ receptors in the dorsal hippocampus rather than the ventral hippocampus might play an essential role in the retrieval of contextual fear memory.

It should also be noted that neither our licking suppression paradigm nor conventional freezing paradigm can discriminate among the deficits in acquisition, consolidation, and retrieval of fear memory in specific cases. Although the conditioned licking suppression paradigm excludes the confounding effects of locomotor activity, this paradigm cannot assess the process of fear acquisition because we cannot observe licking during fear conditioning. Thus, it is impossible to discriminate among the deficits in acquisition, consolidation, and retrieval of fear memory when significant differences in the number of licking between groups appear from the beginning of the test phase. The conventional freezing paradigm can assess the process of fear acquisition, but it would be difficult to interpret the results when significant differences in locomotor activity between groups are observed. Future studies should address these limitations. At least for a while, these two paradigms would be complementary to each other.

The reduction of contextual fear responses in 5-HT_2C_ receptor KO mice might be due to increased BDNF levels in the hippocampus because a previous study demonstrated that BDNF expression in the hippocampus was increased in 5-HT_2C_ receptor KO mice [20]. BDNF Val66Met polymorphism has been associated with impaired memory function, vulnerability to stress, stress-related disorders [26], and cognitive/affective deficits in other psychiatric disorders such as schizophrenia [27]. Thus, it is possible that 5-HT_2C_ receptor KO mice have the resilience to stress by increasing BDNF expression in the hippocampus. Supporting this, 5-HT_2C_ receptor KO mice displayed anxiolytic phenotype [4]. However, the effects of BDNF on fear memory were brain region/timing-dependent and inconsistent among studies [26]. Further studies using conditional KO are required to clarify whether the increased BDNF expression is involved in the reduction of fear responses in 5-HT_2C_ receptor KO mice.

Surprisingly, in a procedure where contextual and cue-specific information where imbedded during conditioning, but tested separately, we found (1) that all differences observed in cued or contextual conditioning were abolished between 5-HT_2C_ receptor KO and WT mice and (2) sustained contextual fear responses over extinction trials. It is worth noting that 5-HT_2C_ receptor KO mice manifested a similar level of contextual fear responses compared to WT mice in this condition, suggesting that the reduction of fear responses observed in 5-HT_2C_ receptor KO mice in the Context-Only condition is less likely to result from a lower sensitivity to the aversive US, and/or a deficit in learning and memory processes. Our results are in line with previous studies in healthy human volunteers showing that a reduction in fear responding to a specific cue is associated with sustained contextual fear responses when contextual and cue-specific information are combined during conditioning, but tested separately [12–14]. Most importantly, the fact that this effect was observed in both KO and WT mice suggests that the 5-HT_2C_ receptor is not necessary for contextual fear expression in our Compound condition. This finding questions the relevance of targeting 5-HT_2C_ receptors for developing therapeutics for PTSD, at least when it comes to exposure-based therapies, and urges caution in the assessment of future candidate drugs.

How a potential interaction between contextual and cue-specific information led to sustained contextual fear responses remains speculative. Associative learning theories state that cue competition occurs when multiple cues are presented simultaneously and followed by a US, a prediction that holds true between cue-specific and contextual information [25, 28]. According to this theoretical framework, cues with the highest saliency become the best predictors of the US, thus provoking the strongest CRs. This phenomenon is well documented in a wide variety of procedures and is referred to as overshadowing [29–31]. It is worth noting that the conditioning phase in both Cued-Only and Compound conditions is the same; that is, both tone-cue and contextual information are conditioned to the US. The main difference between these two conditions resides in the re-exposure of the tone-cue only (i.e., Cued-Only condition) while tone-cue and contextual information are re-exposed in separate phases (i.e., Compound condition). This suggests that the condition of re-exposure following the formation of a traumatic memory is a critical aspect in the effectiveness of re-exposure-based therapies to reduce fear responses.

We cannot exclude the possibility that the reduction of fear responses in 5-HT_2C_ receptor KO mice observed in the Context-Only and Cued-Only conditions could result from the absence of 5-HT_2C_ receptor expression only during a critical period of development. Nonetheless, our results are consistent with a reduction of fear responses observed in adult rats administered intraperitoneally with a 5-HT_2C_ receptor antagonist [6], and with the anxiolytic effect of 5-HT_2C_ receptor antagonism in adult humans [32].

Based on the findings in the present study, we suggest three future directions. First, to improve the screening efficiency of candidate drugs, future studies testing potential therapeutics for PTSD should use a context-cue compound fear conditioning in addition to re-exposing contextual or cued fear-conditioned stimuli separately during extinction. Second, we suggest examining whether the cue-context interaction could be avoided when both elements are exposed simultaneously during the extinction phase. Virtual reality technology appears to be an interesting strategy to tackle this issue in humans and has a potential clinical translational value to patients [33]. Neutralizing context-cue interactions may help in reconsidering the therapeutic efficacy of 5-HT_2C_ receptor antagonists and other drugs that showed promising effects in preclinical studies but failed to pass clinical trials. Third, our data invites mechanistic investigations at both behavioral and neural levels as to how contextual and cued fear conditioning combined within the same procedure bypass anxiolytic-like effects such as those resulting from 5-HT_2C_ receptor gene KO in fear-related responses.

## Supplementary information

Supplementary Figures 1 and 2
