# Supplementary material for: Serotonin 5-HT2C receptor knockout in mice attenuates fear responses in contextual or cued but not compound context-cue fear conditioning
Source: Transl Psychiatry. 2022 Feb 11;12:58. doi: 10.1038/s41398-022-01815-2 (PMC8831648; doi:10.1038/s41398-022-01815-2)
Supplement: Supplementary file 1 — Supplementary Figures 1 and 2 [file 41398_2022_1815_MOESM1_ESM.pptx]

## Slide 1
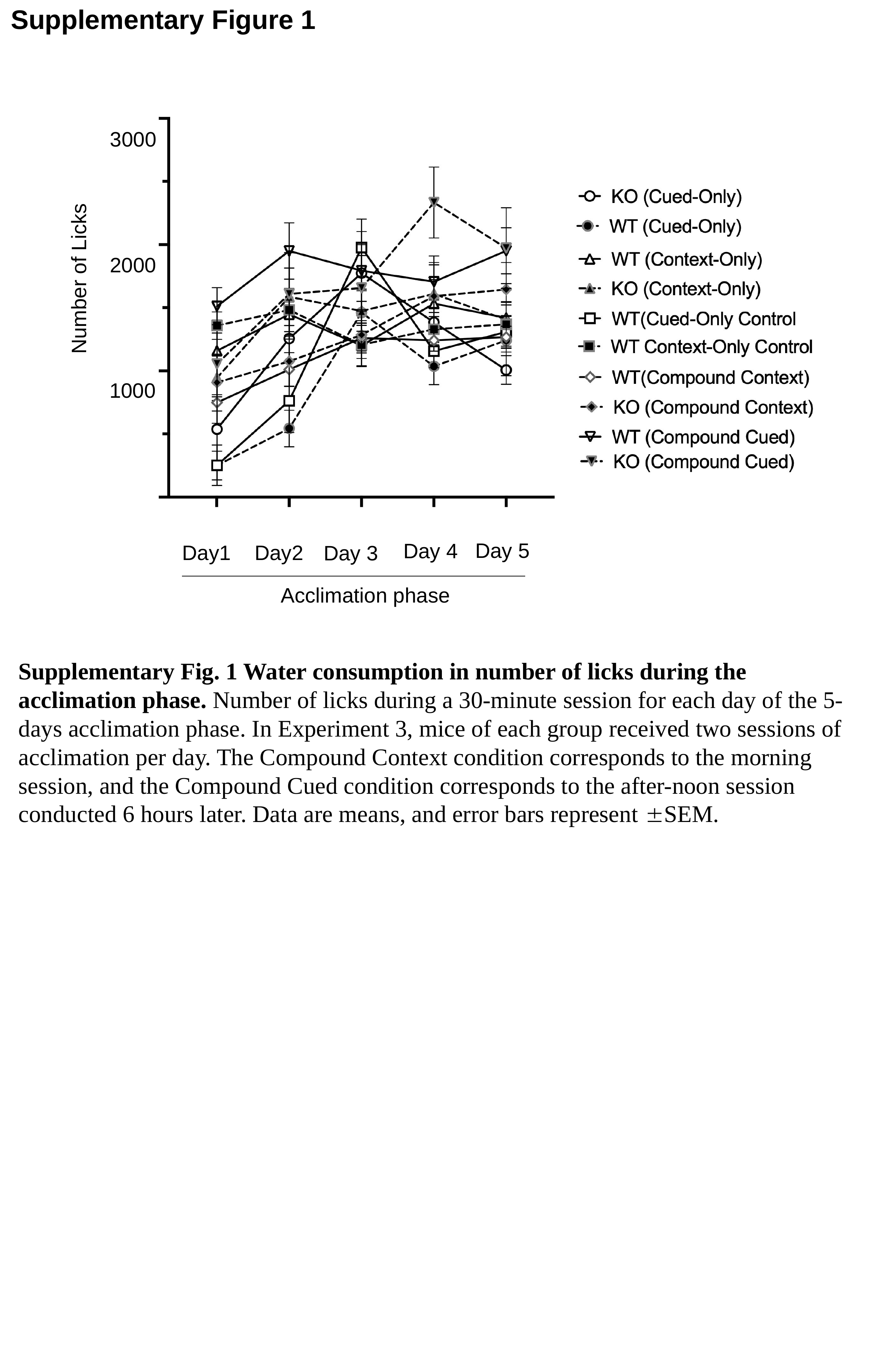

Supplementary Figure 1
3000
2000
Number of Licks
1000
Day 5
Day 4
Day1
Day2
Day 3
Acclimation phase
Supplementary Fig. 1 Water consumption in number of licks during the acclimation phase. Number of licks during a 30-minute session for each day of the 5-days acclimation phase. In Experiment 3, mice of each group received two sessions of acclimation per day. The Compound Context condition corresponds to the morning session, and the Compound Cued condition corresponds to the after-noon session conducted 6 hours later. Data are means, and error bars represent ±SEM.

## Slide 2
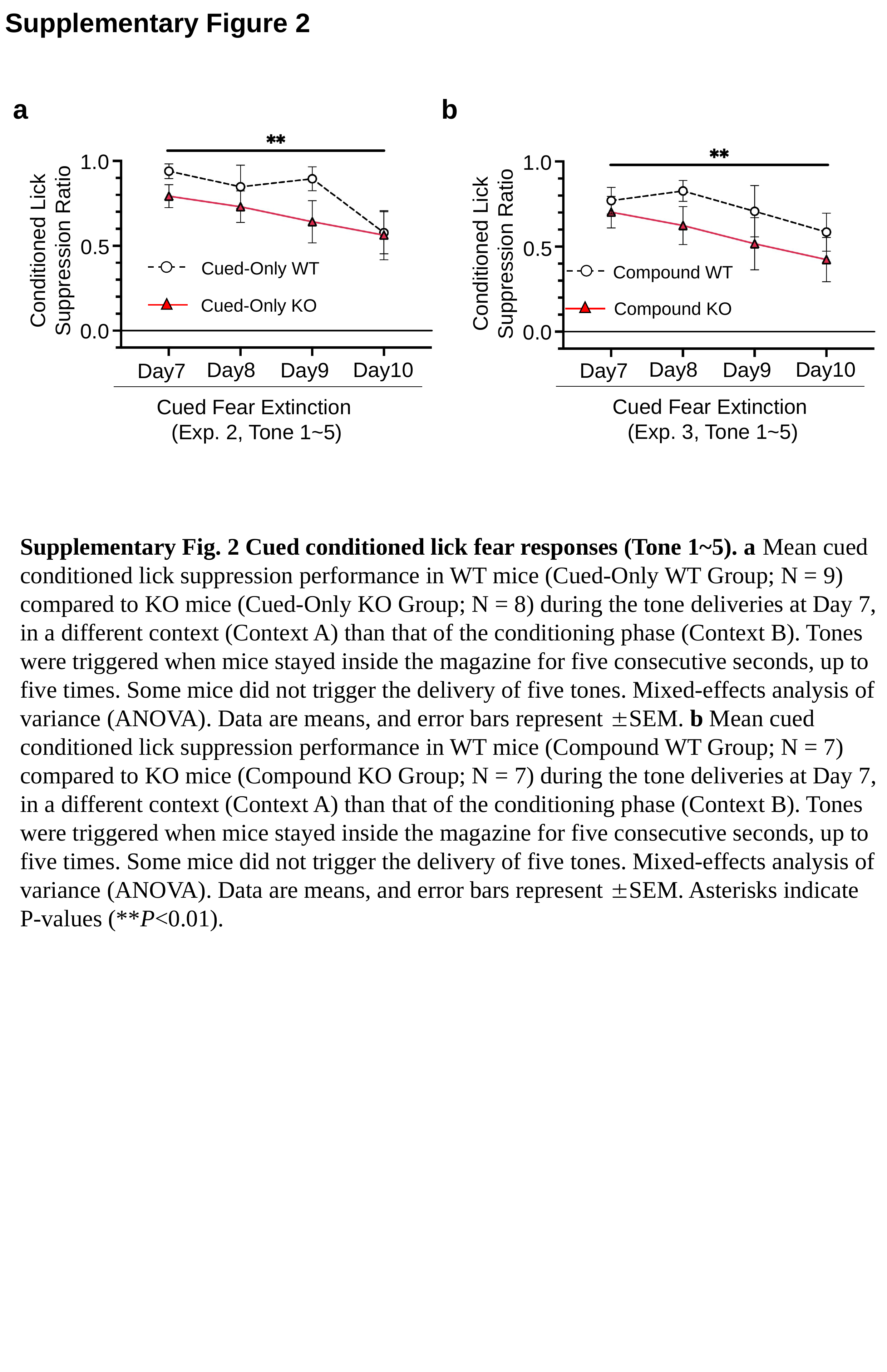

Supplementary Figure 2
a
b
1.0
1.0
Conditioned Lick Suppression Ratio
Conditioned Lick Suppression Ratio
0.5
0.5
Cued-Only WT
Compound WT
Cued-Only KO
Compound KO
0.0
0.0
Day8
Day10
Day9
Day8
Day10
Day9
Day7
Day7
Cued Fear Extinction
(Exp. 3, Tone 1~5)
Cued Fear Extinction
(Exp. 2, Tone 1~5)
Supplementary Fig. 2 Cued conditioned lick fear responses (Tone 1~5). a Mean cued conditioned lick suppression performance in WT mice (Cued-Only WT Group; N = 9) compared to KO mice (Cued-Only KO Group; N = 8) during the tone deliveries at Day 7, in a different context (Context A) than that of the conditioning phase (Context B). Tones were triggered when mice stayed inside the magazine for five consecutive seconds, up to five times. Some mice did not trigger the delivery of five tones. Mixed-effects analysis of variance (ANOVA). Data are means, and error bars represent ±SEM. b Mean cued conditioned lick suppression performance in WT mice (Compound WT Group; N = 7) compared to KO mice (Compound KO Group; N = 7) during the tone deliveries at Day 7, in a different context (Context A) than that of the conditioning phase (Context B). Tones were triggered when mice stayed inside the magazine for five consecutive seconds, up to five times. Some mice did not trigger the delivery of five tones. Mixed-effects analysis of variance (ANOVA). Data are means, and error bars represent ±SEM. Asterisks indicate P-values (**P<0.01).
